# Observed change of the standardized precipitation index, its potential cause and implications to future climate change in the Amazon region

**DOI:** 10.1098/rstb.2007.0022

**Published:** 2008-02-11

**Authors:** Wenhong Li, Rong Fu, Robinson I. Negrón Juárez, Katia Fernandes

**Affiliations:** 1 Earth and Atmospheric Sciences, Georgia Institute of TechnologyFord ES&T Building, 311 Ferst Drive, Atlanta, GA 30332-0340, USA; 2 Department of Ecology and Evolutionary Biology, Tulane UniversityNew Orleans, LA 70118-5698, USA

**Keywords:** standard precipitation index, anthropogenic influence, Amazon, dry events, future climate

## Abstract

Observations show that the standard precipitation index (SPI) over the southern Amazon region decreased in the period of 1970–1999 by 0.32 per decade, indicating an increase in dry conditions. Simulations of constant pre-industrial climate with recent climate models indicate a low probability (*p*=0%) that the trends are due to internal climate variability. When the 23 models are forced with either anthropogenic factors or both anthropogenic and external natural factors, approximately 13% of sampled 30-year SPI trends from the models are found to be within the range of the observed SPI trend at 95% confidence level. This suggests a possibility of anthropogenic and external forcing of climate change in the southern Amazon. On average, the models project no changes in the frequency of occurrence of low SPI values in the future; however, those models which produce more realistic SPI climatology, variability and trend over the period 1970–1999 show more of a tendency towards more negative values of SPI in the future. The analysis presented here suggests a potential anthropogenic influence on Amazon drying, which warrants future, more in-depth, study.

## 1. Introduction

The Amazon rainforests represent the world's most biodiverse ecosystems and play a key role in hydrology, carbon storage and exchange (Malhi & Philip 2005). Seasonal droughts may delay the forest's turnover (Meir & Grace 2005); severe droughts, such as the recent 1998 and 2005 droughts, increase flammability of the forests in part because leaf litter dries and becomes tinder for wildfires (Nepstad *et al.* 2002; Giles 2006). As surface temperatures over the Amazon rise with the increase of global atmospheric CO_2_ (Malhi & Wright 2004; Hansen *et al.* 2006), there are increased concerns about whether drought would become stronger and consequently threaten the rainforests (Cox *et al.* 2004; Malhi *et al.* 2008).

Malhi & Wright (2004) reported that the surface temperatures over the tropical rainforests have increased rapidly since the 1970s, and annual rainfall has decreased over Africa and the south and southeast Asian rainforests. The decrease of annual rainfall over the Amazon is not as clear as that found in other rainforest regions. However, the intensity and frequency of dry anomalies have a stronger influence on the rainforest than does the annual rainfall (Sombroek 2001). Dry anomalies can change without a necessary change in the annual rainfall. Previous studies have used the Palmer drought index (PDI) to examine the global long-term changes of drought including the Amazon (Dai *et al.* 2004). The PDI is intended to represent soil moisture change associated with dry and wet anomalies as determined by observed precipitation and surface temperature and a simple two-layered bucket model. This index has several limitations when applied to rainforests: (i) the PDI was determined by linear fitting of data from a few stations in the central USA, where land surface and vegetation conditions are dominated by short vegetation and crops, substantially different from the tall forests over the Amazon and (ii) the rainforest is more sensitive to dry anomalies during the transition and dry seasons than during the wet season, whereas the PDI is well correlated with and thus largely influenced by cumulative rainfall deficit on a 12-month time scale (McKee *et al.* 1995). In comparison, the standard precipitation index (SPI) is designed to flexibly present the incremental rainfall deficit, the source of drought, at any time scale of interest (McKee *et al.* 1993). For the Amazon, SPI for the six-month time scale would allow us to distinguish incremental dry anomalies between dry and wet seasons. Thus we will examine the SPI over a six-month time scale in this study.

This paper aims to determine whether the SPI has changed in the late twentieth century. If so, is the change mainly caused by natural climate variability or due to anthropogenic forcing? Finally, we will examine whether or not the SPI in the twenty-first century would indicate more droughts with an increase of anthropogenic forcings as determined by the simulations of phase 3 of the Coupled Model Intercomparison Project (CMIP3) participating in the Intergovernmental Panel for Climate Change Fourth Assessment (IPCC AR4). We apply a climate detection and attribution analysis using ensemble simulations from multiple climate models following Santer *et al.* (2007). A similar analysis has been used widely to discern ‘anthropogenic influence’ on surface temperature warming and changes in the atmospheric column total moisture and rainfall (Mitchell *et al.* 2001; Santer *et al.* 2003, 2007; Hoerling *et al.* 2006).

## 2. Data and methods

Physically, the SPI represents the total difference of precipitation for a given period of time (six-month period for this study) from its climatological mean value and then normalized by the precipitation standard deviation for the same six months in the climatological annual cycle computed using data over the entire period of the analysis. Thus dry events are represented by negative values of SPI. The SPI value is determined by the probability of a given incremental rainfall anomaly occurring during the period of the analysis based on a gamma probability density function fit to the time series of monthly precipitation. For example, a SPI value of −1.5 presents a dry anomaly that occurred with a probability of 6.7% or less (McKee *et al.* 1993) during the analysis period, and thus falls into the severe drought category in the SPI ranking system. SPI has been widely used to monitor drought (negative SPI) or wet spells (positive SPI) in the USA over multi-decades (Heim 2002).

The monthly precipitation dataset used to derive SPI is the University of East Anglia Climatic Research Unit global dataset (New *et al.* 1999; hereafter referred to as CRU dataset). CRU precipitation started in 1901 and ended in 2002. We use the last 30-year SPI trends for the period of 1970–1999 due to lack of an adequate rain gauge network in the Amazon prior to 1970 (Hulme 1995) and to match the 1999 ending date for most of the twentieth-century simulations by the CMIP3 climate models. Since previous studies suggest that the surface temperature warming over the tropical forests started mainly after the mid-1970s (Malhi & Wright 2004), the period we chose should capture the possible rainfall changes associated with surface warming. In addition, the trend of the SPI over the 30 years is similar to that over the period of 1951–1999 (not shown here), suggesting that our result is not sensitive to the detailed period we chose.

Precipitation from the coupled ocean-atmospheric general circulation models for the IPCC AR4 that is available at the Program for Climate Model Diagnosis and Intercomparison (PCMDI) is used in the study. We considered three sets of the simulations: pre-industrial control runs (PICNTRL), twentieth-century forced runs (20C3M) and the twenty-first-century simulations under the emission scenario A1b. There are 16 modelling groups in the PICNTRL, the twentieth-century forced runs and the twenty-first-century simulations (see electronic supplementary material for details). Five modelling groups provide precipitation results for at least two different model configurations in the twentieth-century control and forced runs. Results from 20 and 23 different versions of climate models were analysed for the PICNTRL and 20C3M simulations, respectively.

The PICNTRL represents natural variability of a coupled ocean and atmosphere climate system with greenhouse gases fixed at the pre-industry level (e.g. CO_2_ concentration is approx. 270–280 ppm). The 20C3M simulations are driven by the same estimated anthropogenic forcings of long-lived greenhouse gases as for the twentieth century but with different natural external forcings and other radiative agents such as solar irradiance variation, volcanic aerosols, sulphate and black carbon aerosols, and land use. The twenty-first-century simulations are driven by anthropogenic forcing under the A1b scenario, which projects a CO_2_ concentration increase from 370 ppm in 2000 to 720 ppm in 2100. We use the comparison between the PICNTRL and the 20C3M simulations to examine the influence of anthropogenic and external forcings versus that of natural climate variability on the SPI changes. We also compare the 20C3M simulations with and without natural external forcings to assess the relative influences of the anthropogenic forcing due to the long-lived greenhouse gases versus the forcing due to other anthropogenic radiative agents and external natural forcing on the changes of SPI. The differences between these two groups of 20C3M are influenced by the differences in both forcings and physics between models. Finally, we compare the distributions of SPI trends in the A1b simulations to those of 20C3M to explore the changes of the dry events in the future.

The state-of-art attribution studies apply the optimal fingerprinting technique to identify the global spatial and temporal patterns due to slowly increasing anthropogenic forcing with minimum influence of natural variability. At the regional scale and with relatively coarse spatial resolutions of the CMIP3 climate models, we cannot expect a reliable detection of the spatial pattern. Thus we will focus on temporal changes of the area averaged SPI over the southern Amazon region (5° S–15° S, 75° W–45° W; Li *et al.* 2006) using the approach of Santer *et al.* (2007). This approach uses multiple models with different resolution, physics, parametrization and forcing to determine the robustness of the SPI changes under different climate scenarios. Simulations by the 20 PICNTRL models yield 83 independent samples of non-overlapping 30-year SPI trends, and those by the 23 20C3M models yield 243 independent samples of non-overlapping 30-year SPI trends. The distributions of the SPI trends derived from these two climate scenarios are compared with the observed SPI trends for the period of 1970–1999 to determine the extent to which natural climate variability and anthropogenic and external natural forcing can explain the observed change in SPI.

The SPIs from both CRU monthly precipitation data and from the models are calculated following McKee *et al.* (1993). We first test whether the twentieth-century simulations forced by historical changes of the anthropogenic and external natural forcing can adequately reproduce the observed mean, variability and trend of the observed SPI. Then, we examine whether the model estimates of internal climate variability without any changes in external forcing or changes in anthropogenic and external forcing can better explain the observed SPI trend in the period of 1970–1999. Finally, we will examine the projected changes of the SPI under the A1b scenario and examine how the realism of the climate models, as estimated by comparison to the observations during the period of 1970–1999, would impact the projected changes.

## 3. Results

Figure 1 shows the differences in the frequency, mean values, as well as trends of SPI between the 20C3M simulations and observations for the period of 1970–1999. Figure 1*a* shows that 17 climate models agree with observations within ±10% of the observed frequency of dry events (dashed lines in figure 1*a*). Figure 1*b* indicates that the same 17 model simulations also realistically simulate the mean value of the negative SPI (figure 1*b*). The same sign of SPI trend (figure 1*c*) as observed is shown in 11 out of these 17 models. These 11 models are thus referred to as the ‘good’ models for the 20C3M simulations. Here good models for the present climate imply that they are also going to be good for the future.

Figure 2 compares the observed SPI trend with those trends derived from the unforced PICNTRL simulations (figure 2*a*) and externally forced 20C3M simulations (figure 2*b*). The Mann–Kendall statistical test (Hirsch *et al.* 1982) showed that the observed decrease in SPI value by approximately 0.32 per decade obtained from CRU precipitation data is significant. We also examined the SPI trends using different precipitation data including the VASClimO-50yr precipitation climatology (Beck *et al.* 2005; hereafter referred to as VASClimO) and Climate Prediction Center improved gridded historical daily precipitation analysis for Brazil starting from 1979 (Silva *et al.* 2007; hereafter referred to as Silva-data). The VASClimO data suggest a significant SPI trend of −0.38 per decade and the Silva-data suggest a significant SPI trend of −0.49 per decade. Thus all three rainfall datasets consistently suggest a significant decrease of the SPI during the late twentieth century.

The distribution of the SPI trends obtained from PICNTRL runs shows that approximately 32% of the cases are less than −0.1 per decade, 50% greater than 0.1 per decade and 18% without trends (figure 2*a*). None of the modelled trends are within the range of observed SPI trends at 95% confidence level. The bias towards a positive SPI trend may be due to insufficient samples for the PICNTRL simulations. Because the standard deviations of observed SPI (1.0016) and modelled SPI (from 0.8925 to 1.1163) are comparable, it is unlikely that all the PICNTRL simulations would underestimate the natural climate variability. Thus figure 2*a* suggests that the observed change in the SPI exceeds the range of the natural climate variability.

When model experiments are forced by anthropogenic and natural external forcings, approximately 13% of the samples are within the range of observed SPI trend at 95% confidence level. The trend distributions shift systematically towards the negative values (figure 2*b*), i.e. approximately 51% of the samples show negative trends of up to −0.1 per decade, 24% no trends and 25% show positive trends of 0.1 per decade or above. Thus a greater fraction of the trends obtained from forced runs agree with the observed SPI trends compared with the natural climate variability simulations (PICNTRL).

Could external natural forcing (e.g. solar variability and volcanic aerosols) be responsible for the observed SPI trend? To answer this question, figure 2*b* compares the distributions of SPI trends provided by two groups of 20C3M simulations. The first group consists of 102 samples obtained by simulations driven only by the anthropogenic forcing, and the second group consists of 141 samples of SPI trends obtained from the simulations driven by both the anthropogenic forcing and natural external forcings. There are no clear differences in the SPI trends between these two groups of 20C3M simulations, although the negative SPI trends driven only by the anthropogenic forcing (figure 2*b*) are approximately 2% stronger than those forced by both anthropogenic forcing and natural external forcings (figure 2*b*). Thus the negative SPI trends in the 20C3M simulations do not appear to depend on the presence of external natural forcing. Approximately 24% of the samples from the 11 good models are within the range of observed SPI trend at 95% confidence level. The 11 good models also show approximately 25% more negative SPI trends than those obtained from all 23 20C3M simulations. Overall, figure 2 suggests that the observed negative SPI trend (−0.32 per decade) can be better explained by the simulations with the anthropogenic and external forcings than by the natural variability of the climate system.

Figure 3 shows the projected distribution of the SPI trends in the twenty-first century after a continuous increase of CO_2_. The population of SPI trends from all of the 23 model simulations (A1b) peaks at zero, with 13% fewer negative SPI trends and similar population of positive trends in the twenty-first century compared with those in the twentieth century. However, for the 11 good models as determined in figure 1 based on the observations in the twentieth century, the distribution of the SPI trends systematically shifts towards negative SPI trends (67%), with 15% no trend and 18% positive SPI trends. These good models project an increase of approximately 16% dry events over the southern Amazon in the twenty-first century from the twentieth century. The wider spread of the SPI trends in the twenty-first century (like SPI trends that are less than −0.5 per decade) also suggests the possibility of stronger and more extreme dry events in the future than in the twentieth century. The difference in climate projections between all the climate models and the so-called ‘good’ models illustrates the importance of ranking the climate projections based on realism of the models.

## 4. Conclusions

Observations show a significant negative trend in the SPI (−0.32 per decade), a widely used drought index, over the southern Amazon region during the period of 1970–1999, although the mechanism is unknown. The observed negative SPI trend cannot be reproduced by the ensemble simulations of 20 CMIP3 climate models with the fixed pre-industrial concentrations of greenhouse gases. However, approximately 13% of the ensemble simulations mostly by the same group of models (23 models) forced by the prescribed anthropogenic and external forcings for the twentieth century show agreement with the observed SPI trend. The agreement increases to 24% for the climate models that realistically reproduce the frequency, mean and climate variability of the SPI. These results suggest a possible anthropogenic cause for the increase of dry events over the Amazon region in the late twentieth century. For the twenty-first century, those models realistically simulating the changes of the SPI in the twentieth century suggest an overall shift of the SPI towards more frequent and/or intense dry events, and probably stronger extreme dry events over the Amazon as anthropogenic forcing continues to increase.

## Figures and Tables

**Figure 1:**
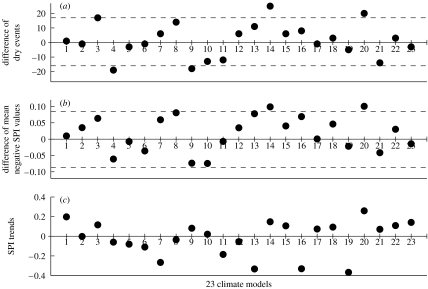
(*a*) The difference between the modelled and observed dry events, i.e. negative SPI values, during the period of 1970–1999. Horizontal lines denote within ±10% of the observed SPI values for dry events; (*b*) as in (*a*) but for the differences in mean negative SPI between the models and observation data; horizontal lines denote ±10% of the observed mean negative SPI; (*c*) changes of SPI value per decade during the period of 1970–1999 (*x*-axis represents 23 climate models of the 20C3M simulations).

**Figure 2:**
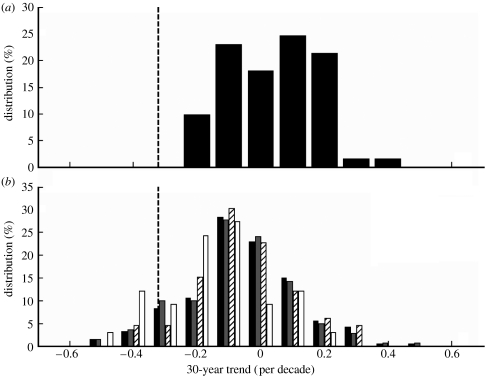
(*a*) The comparison between the observed SPI trend (dashed line) and the trends observed from the PICNTRL simulations. The bars show fractional distribution of SPI trends obtained from the PICNTRL simulations; (*b*) same as (*a*) but for the SPI trends obtained from the 20C3M model simulations. The black, hatched, grey and white bars represent the distribution of the SPI trends for all 20C3M simulations, simulations driven by anthropogenic forcing only, by both anthropogenic and natural external forcing (solar irradiance and volcanic aerosols) and by the 11 ‘good’ models, respectively (*x*-axis represents 30-year trend of the SPI value change per decade and *y*-axis represents percentage of occurrences for each bin relative to the total number of samples).

**Figure 3:**
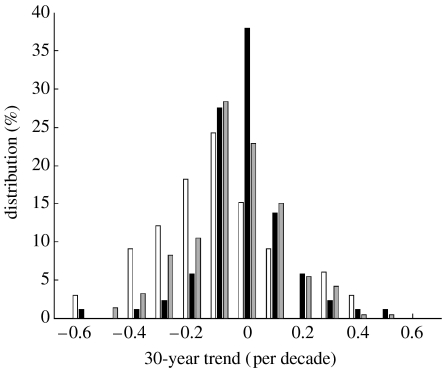
The distributions of SPI trends in the twenty-first-century simulation (A1b) obtained from all the 23 models (black) and the 11 ‘good’ models (white) compared with those of the twentieth-century simulations (20C3M, grey) (*x*-axis represents 30-year trend of the SPI value change per decade and *y*-axis represents percentage of occurrences for each bin relative to the total number of samples).

## Supplementary Material

Supplementary Material The supplementary materials list all CMIP3 modelling groups participating in IPCC AR4. The precipitation output from these climate models are used to calculate the SPI in the study.
