# Supplementary material for: Observed change of the standardized precipitation index, its potential cause and implications to future climate change in the Amazon region
Source: Philos Trans R Soc Lond B Biol Sci. 2008 Feb 11;363(1498):1767–72. doi: 10.1098/rstb.2007.0022 (PMC2373881; doi:10.1098/rstb.2007.0022)
Supplement: Supplementary file 1 [file rstb20070022s01.doc]

Observed Change of the Standardized Precipitation Index, Its Potential Cause and Implications to Future Climate in the Amazon Region

Wenhong Li^1^, Rong Fu^1^, Robinson I. Negrón Juárez^2^, and Katia Fernandes^1^

^1^Earth and Atmospheric Sciences, Georgia Institute of Technology, Atlanta, Georgia

^2^Ecology and Evolutionary Biology, Tulane University, New Orleans, Louisiana

## Revision for ****Philosophical Transactions of the Royal Society****

November 26, 2007

Corresponding author address: Dr. Wenhong Li, Earth and Atmospheric Sciences, Georgia Institute of Technology, Ford ES&T Bldg., 311 Ferst Drive, Atlanta, GA 30332-0340; (404) 894-1624; Fax: (404) 894-5638; E-mail: [wenhong@eas.gatech.edu](mailto:wenhong@eas.gatech.edu)

**Supporting Text: Modeling groups contributing to IPCC AR4**

1. Hadley Centre for Climate Prediction and Research / Met Office, UK, [UKMO-HadCM3, UKMO-HadGEM1]

2. Bjerknes Centre for Climate Research, Norway, [BCCR-BCM2.0]

3. National Center for Atmospheric Research, USA, [CCSM3, PCM]

4. Canadian Centre for Climate Modelling & Analysis, Canada, [CGCM3.1(T47), CGCM3.1(T63)]

5. Météo-France / Centre National de Recherches Météorologiques, France, [CNRM-CM3]

6. CSIRO Atmospheric Research, Australia, [CSIRO-Mk3.0]

7. Max Planck Institute for Meteorology, Germany, [ECHAM5/MPI-OM]

8. Meteorological Institute of the University of Bonn, Meteorological Research Institute of KMA, and Model and Data group, Germany / Korea, [MIUB/ECHO-G]

9. LASG / Institute of Atmospheric Physics, China, [FGOALS-g1.0]

10. US Dept. of Commerce / NOAA / Geophysical Fluid Dynamics Laboratory, USA, [GFDL-CM2.0, GFDL-CM2.1]

11. NASA / Goddard Institute for Space Studies, USA, [GISS-AOM, GISS-EH, GISS-ER]

12. Instituto Nazionale di Geofisica e Vulcanologia, Italy, [INGV-SXG]

13. Institute for Numerical Mathematics, Russia, [INM-CM3.0]

14. Institut Pierre Simon Laplace, France, [IPSL-CM4]

15. Center for Climate System Research (The University of Tokyo), National Institute for Environmental Studies, and Frontier Research Center for Global Change (JAMSTEC), Japan, [MIROC3.2(hires), MIROC3.2(medres)]

16. Meteorological Research Institute, Japan, [MRI-CGCM2.3.2]
